# Supplementary material for: A Humanized Yeast Phenomic Model of Deoxycytidine Kinase to Predict Genetic Buffering of Nucleoside Analog Cytotoxicity
Source: Genes (Basel). 2019 Sep 30;10(10):770. doi: 10.3390/genes10100770 (PMC6826991; doi:10.3390/genes10100770)
Supplement: Supplementary file 1 [file genes-10-00770-s001.zip › Additional_Files/Additional_File7_GOTermHeatmaps.zip!/Additional_File7_GOTermHeatmaps/postreplication_repair.pdf]

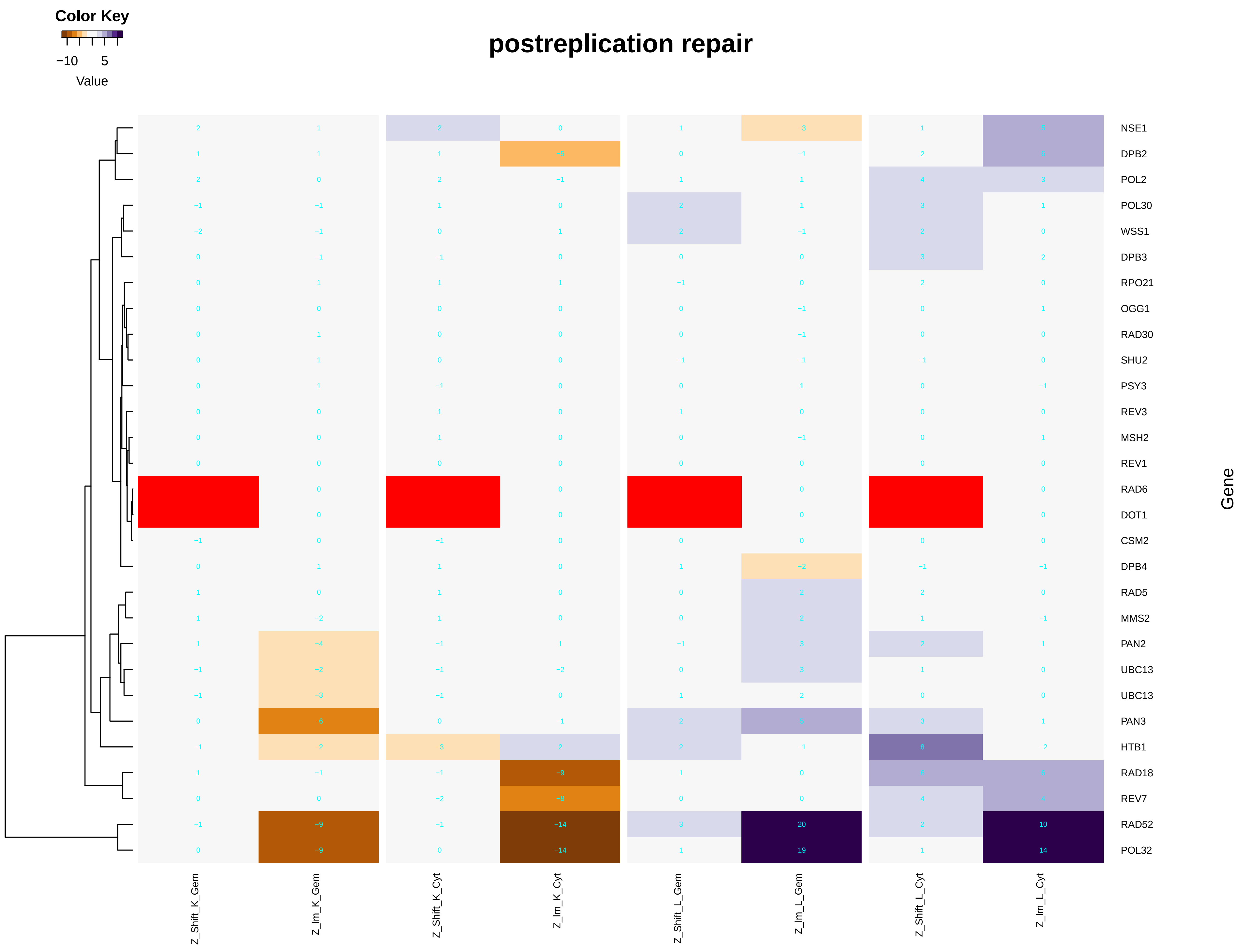

postreplication repair
Color Key
−10
5
Value
NSE1
DPB2
POL2
POL30
WSS1
DPB3
RPO21
OGG1
RAD30
SHU2
PSY3
REV3
MSH2
REV1
RAD6
DOT1
CSM2
DPB4
RAD5
MMS2
PAN2
UBC13
UBC13
PAN3
HTB1
RAD18
REV7
RAD52
POL32
Gene
Z_Shift_K_Gem
Z_Im_K_Gem
Z_Shift_K_Cyt
Z_Im_K_Cyt
Z_Shift_L_Gem
Z_Im_L_Gem
Z_Shift_L_Cyt
Z_Im_L_Cyt

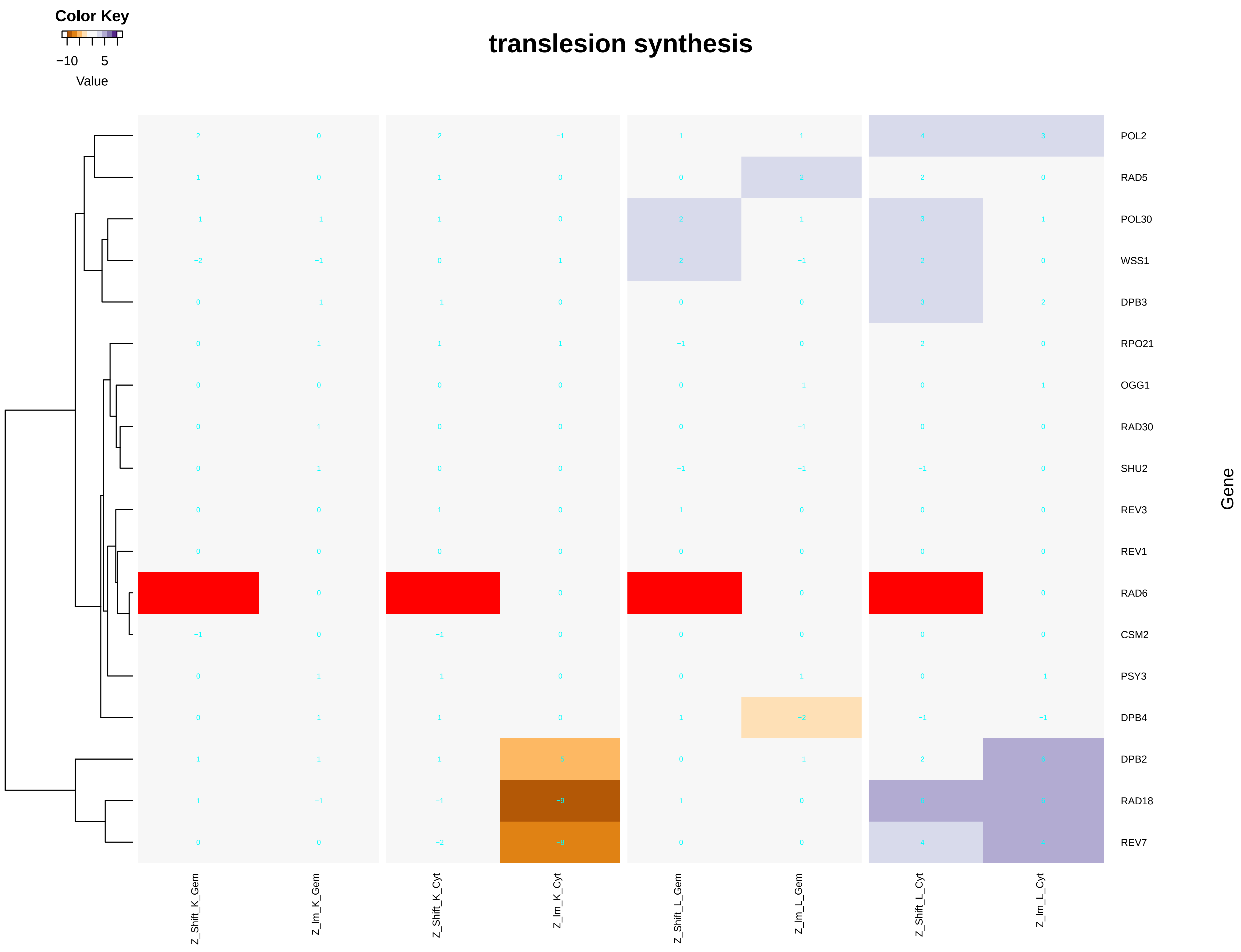
Color Key
−10 5
Value
translesion synthesis
POL2
RAD5
POL30
WSS1
DPB3
RPO21
OGG1
RAD30
SHU2
REV3
REV1
RAD6
CSM2
PSY3
DPB4
DPB2
RAD18
REV7
Gene
Z_Shift_K_Gem
Z_Im_K_Gem
Z_Shift_K_Cyt
Z_Im_K_Cyt
Z_Shift_L_Gem
Z_Im_L_Gem
Z_Shift_L_Cyt
Z_Im_L_Cyt

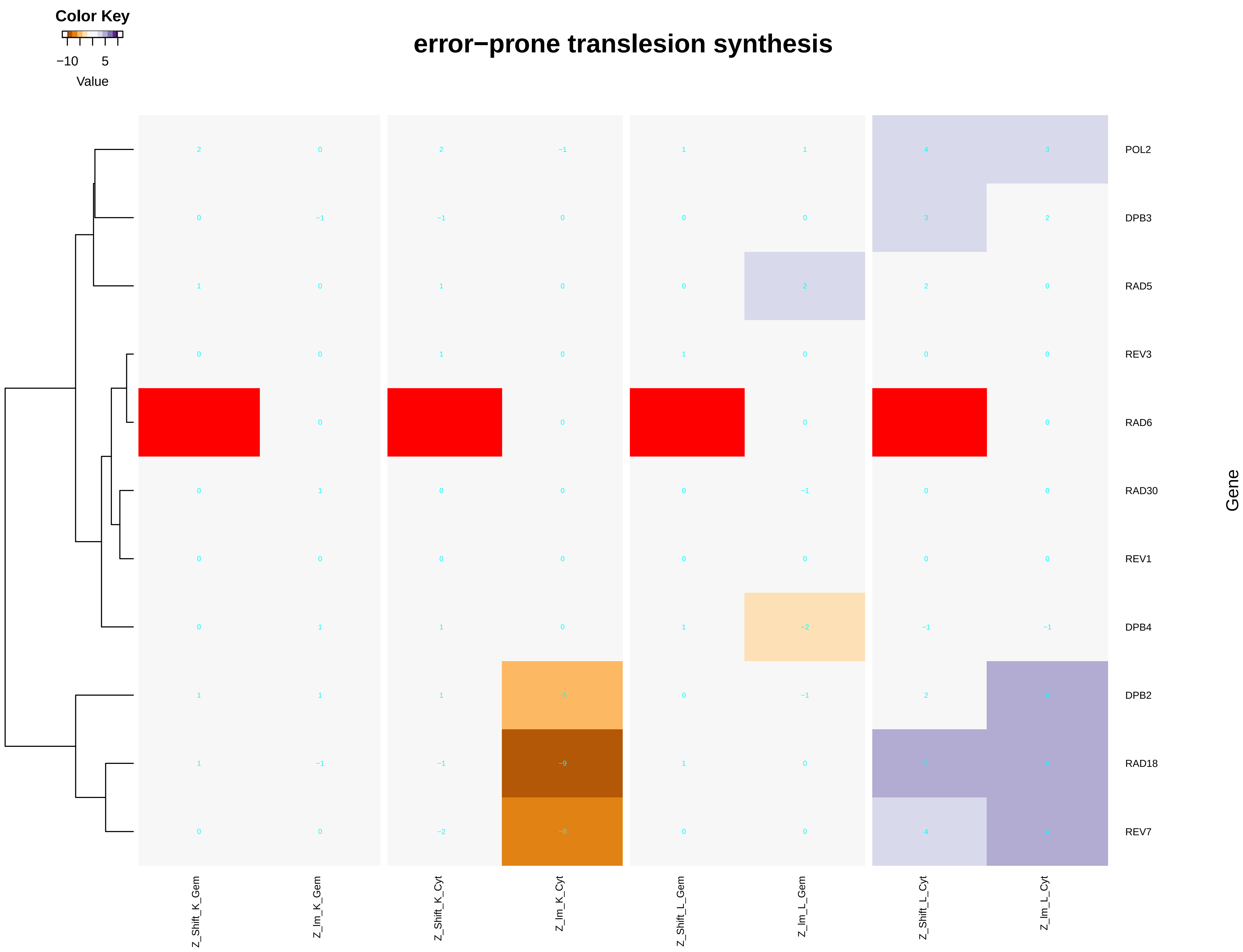

Color Key
−10
5
Value
error−prone translesion synthesis
POL2
DPB3
RAD5
REV3
RAD6
RAD30
REV1
DPB4
DPB2
RAD18
REV7
Gene
Z_Shift_K_Gem
Z_Im_K_Gem
Z_Shift_K_Cyt
Z_Im_K_Cyt
Z_Shift_L_Gem
Z_Im_L_Gem
Z_Shift_L_Cyt
Z_Im_L_Cyt

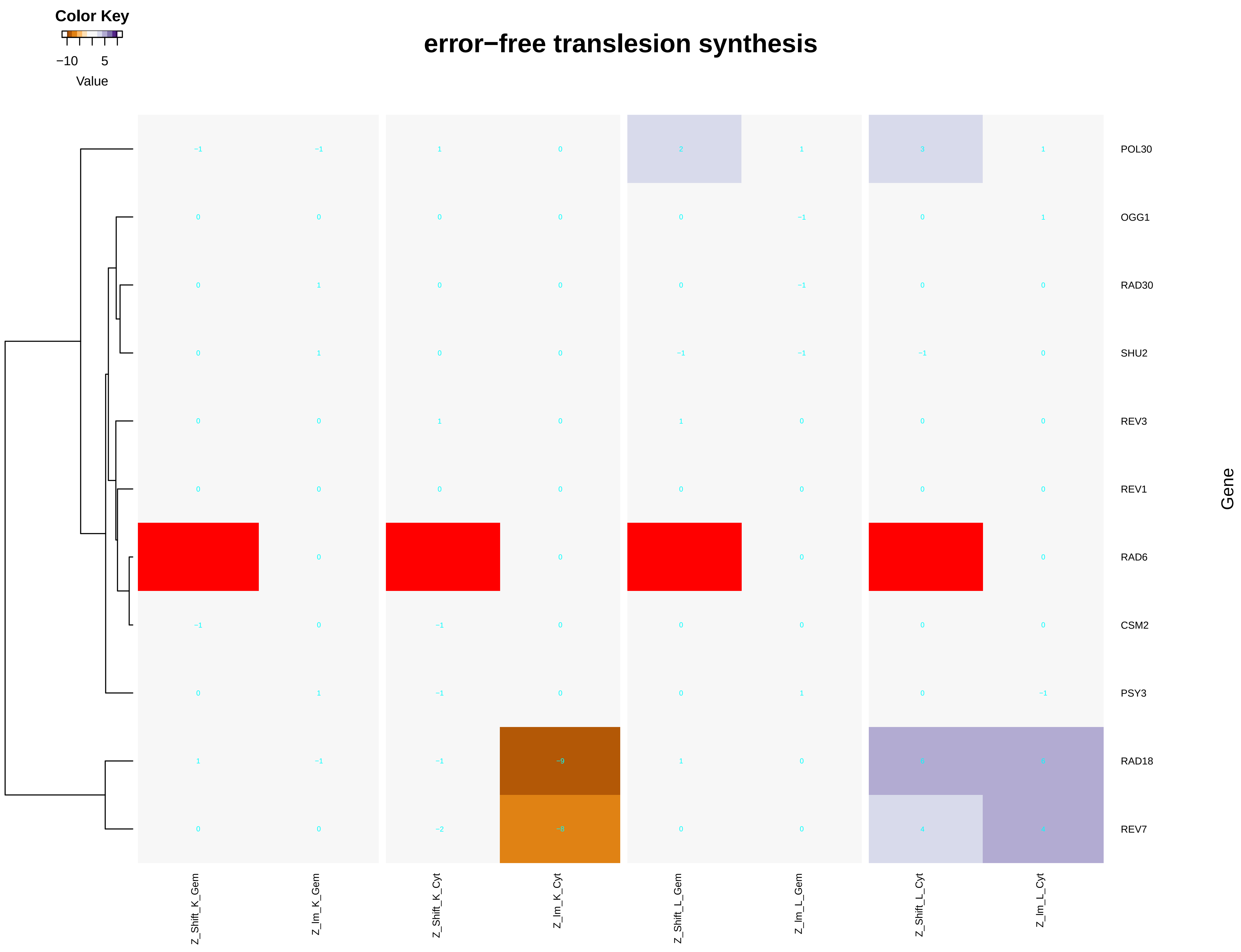

error−free translesion synthesis
Color Key
−10
5
Value
POL30
OGG1
RAD30
SHU2
REV3
REV1
RAD6
CSM2
PSY3
RAD18
REV7
Gene
Z_Shift_K_Gem
Z_Im_K_Gem
Z_Shift_K_Cyt
Z_Im_K_Cyt
Z_Shift_L_Gem
Z_Im_L_Gem
Z_Shift_L_Cyt
Z_Im_L_Cyt
